# Temporal structure in spiking patterns of ganglion cells defines perceptual thresholds in rodents with subretinal prosthesis

**DOI:** 10.1038/s41598-018-21447-1

**Published:** 2018-02-16

**Authors:** Elton Ho, Henri Lorach, Georges Goetz, Florian Laszlo, Xin Lei, Theodore Kamins, Jean-Charles Mariani, Alexander Sher, Daniel Palanker

**Affiliations:** 1 Department of Physics, Stanford University, Stanford, CA 94305 USA; 2 Hansen Experimental Physics Laboratory, Stanford University, Stanford, CA 94305 USA; 3 Department of Ophthalmology, Stanford University, Stanford, CA 94305 USA; 4 Department of Neurosurgery, Stanford University, Stanford, CA 94305 USA; 5 Department of Electrical Engineering, Stanford University, Stanford, CA 94305 USA; 6 Santa Cruz Institute for Particle Physics, University of California, Santa Cruz, CA 95064 USA

## Abstract

Subretinal prostheses are designed to restore sight in patients blinded by retinal degeneration using electrical stimulation of the inner retinal neurons. To relate retinal output to perception, we studied behavioral thresholds in blind rats with photovoltaic subretinal prostheses stimulated by full-field pulsed illumination at 20 Hz, and measured retinal ganglion cell (RGC) responses to similar stimuli *ex-vivo*. Behaviorally, rats exhibited startling response to changes in brightness, with an average contrast threshold of 12%, which could not be explained by changes in the average RGC spiking rate. However, RGCs exhibited millisecond-scale variations in spike timing, even when the average rate did not change significantly. At 12% temporal contrast, changes in firing patterns of prosthetic response were as significant as with 2.3% contrast steps in visible light stimulation of healthy retinas. This suggests that millisecond-scale changes in spiking patterns define perceptual thresholds of prosthetic vision. Response to the last pulse in the stimulation burst lasted longer than the steady-state response during the burst. This may be interpreted as an excitatory OFF response to prosthetic stimulation, and can explain behavioral response to decrease in illumination. Contrast enhancement of images prior to delivery to subretinal prosthesis can partially compensate for reduced contrast sensitivity of prosthetic vision.

## Introduction

Retinal degenerative diseases are the leading cause of untreatable blindness. Age-related macular degeneration alone affects 8.7% of population worldwide, and the number is projected to reach 196 million by 2020 and 288 million by 2040^1^. During retinal degeneration, patients gradually lose photoreceptors, while the inner retinal neurons survive to a large extent^2–4^, albeit with some rewiring over time^5^. Retinal prostheses aim to restore sight by reintroducing visual information using electrical stimulation of the surviving retinal neurons. Epiretinal prostheses typically target the retinal ganglion cells (RGC)^6^, although improved phosphene localization has been reported with activation of the inner retina using longer pulses^7^. Subretinal implants stimulate the inner retinal neurons (primarily bipolar cells), and rely on the retinal network to transmit their responses to RGCs^8^. Both types of implants have been approved for clinical testing^9,10^, but current systems are powered by extra-ocular electronics, and therefore require trans-scleral cables and complex surgeries.

We developed a completely wireless approach based on subretinally placed photodiode arrays, which photovoltaically convert projected light patterns into local electric current in each pixel. Images captured by the camera are processed and projected onto the retina from video goggles using near-infrared (NIR, 880–915 nm) light to avoid photophobic and phototoxic effects of bright illumination^11^. Current flowing through the retina between the active and return electrodes stimulates the nearby inner retinal neurons^8,12^. Electrical stimulation requires charge-balanced pulses to avoid irreversible electrochemical reactions at the electrode-electrolyte interface. Previously we demonstrated retinal adaptation to high frequency (>20 Hz) network-mediated stimulation^13,14^, as opposed to direct stimulation of ganglion cells, which can follow stimuli at rates exceeding 200 Hz^15^. Adaptation to subretinal stimulation at high frequencies shares similarities with naturally occurring flicker fusion in normal vision, which allows continuous perception of movies composed of static frames.

Clinically, patients implanted with subretinal prostheses report patterned percepts of light, called phosphenes, following electrical activation of the inner retinal layer^9,16^ but little is known about the neural mechanisms mediating perception of prosthetic vision. To relate retinal output to perception, we recorded firing patterns of the retinal ganglion cells *ex-vivo* using a 512-channel multielectrode array recording system (MEA)^17^, and examined behavioral responses in blind Royal College of Surgeons rats implanted with a retinal prosthesis using the animal’s startling response to various stimuli. Using a carrier frequency above flicker fusion to deliver trains of electrical activation to the inner nuclear layer, perceptual brightness can be slowly modulated by adjusting either the amplitude or duration of the pulsed stimuli. In a previous *ex-vivo* study we measured an increase in spiking rate with brighter illumination, and the corresponding sensitivity threshold to full-field changes in illumination was about 60%, as opposed to 2–3% with natural vision in rodents^18^. Here, we performed behavioral measurements of the temporal contrast sensitivity using the same kind of implants in rats, and observed much lower threshold than suggested by previous *ex-vivo* analysis. We then revisited the measurements of contrast sensitivity *ex-vivo* using a novel analysis of the firing patterns, rather than simple spike counting, and observed contrast sensitivity in closer agreement with behavioral measurements.

## Results

### Behavioral response to electrical stimulation

**Figure 1:**
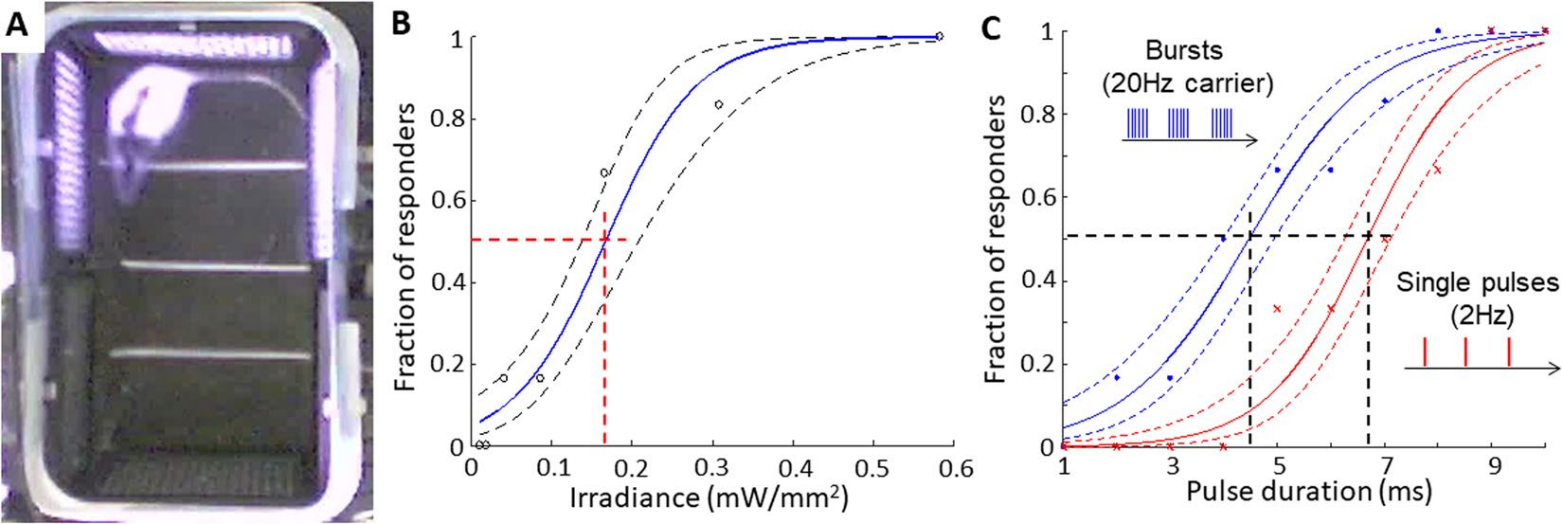
Behavioral determination of the stimulation thresholds. (**A**) RCS rat in a cage surrounded by NIR (850 nm) LED panels. (**B**) Probability of the startling response to 10 ms pulses at 2 Hz, as a function of light intensity. (**C**) Probability of the startling response to stimulation at 0.58 mW/mm^2^ irradiance with single pulses at 2 Hz and with 2 s bursts at 20 Hz, as a function of pulse duration. Confidence interval of one standard deviation is shown with dash lines. Black lines indicate the ED50 threshold values.

Stimulation thresholds and sensitivity of prosthetic vision to pulsed full-field illumination were assessed using a startling response (freezing behavior) in Long Evans (LE, n = 3) and Royal College of Surgeons rats (RCS, n = 6) with subretinally implanted photovoltaic arrays (see Methods). Animals were placed in a cage with sidewalls consisting of LED panels providing a homogeneous 850 nm illumination at 0.58 mW/mm^2^ (Fig. 1A). The implanted RCS rats responded to low frequency stimulation (2 Hz, 10 ms pulses, 0.58 mW/mm^2^) by startling, similar to fear-conditioned^19^ or auditory-evoked freezing^20^. Prior to NIR stimulus, the rats moved around the cage freely; when the stimulus was applied, the rats immediately stopped their movement for a few seconds, including nose and whiskers (see Suppl. Movie 1). Non-implanted animals did not respond to photovoltaic stimulation at any settings, confirming that the response was elicited by the implant rather than by perception of the infrared light. Normally sighted rats (LE) with photovoltaic implants did not respond to stimulation either, suggesting that normal vision in our experimental paradigm (room lights on) dominates the small prosthetic input^21^, and thereby eliminates the startling response observed in blind animals lacking any additional visual information.

Stimulation thresholds were defined independently for irradiance and for pulse duration, as the 50% probability of eliciting a response (ED50). As shown in Fig. 1B,C, stimulation thresholds were 0.17 ± 0.04 mW/mm^2^ for 10 ms pulses, and 6.7 ± 0.6 ms for 0.58 mW/mm^2^ irradiance.

### Behavioral response thresholds to temporal contrast steps with prosthetic stimulation

**Figure 2:**
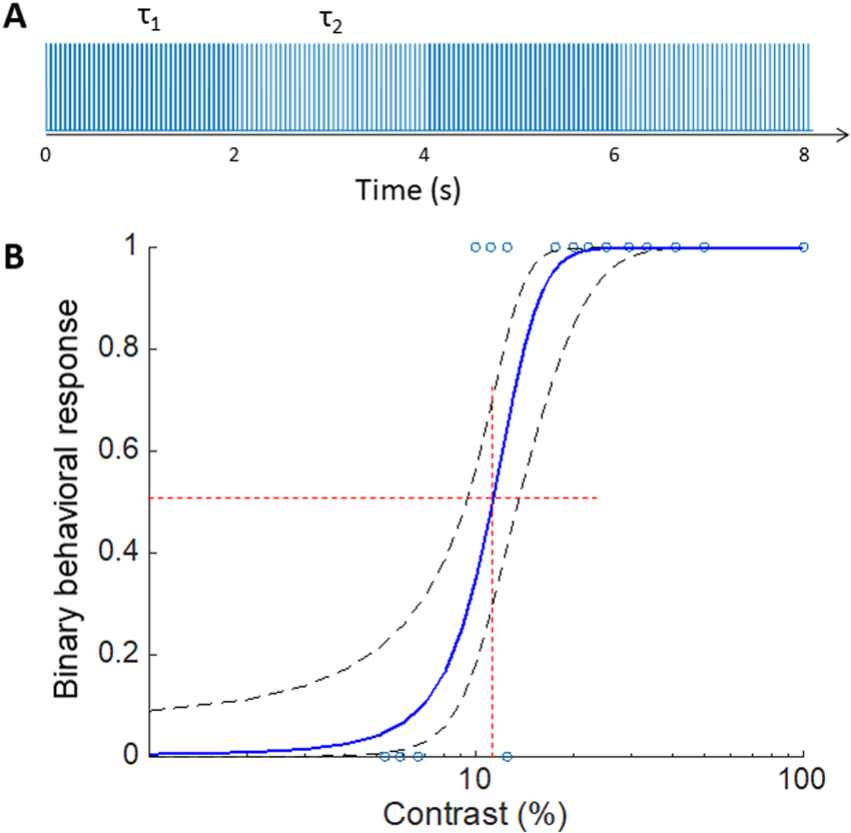
Contrast sensitivity measurement *in-vivo*. (**A**) Light intensity (radiant exposure) of the LED panels was modulated in 2-second-long steps by adjusting duration of the pulses, repeated at 20 Hz. (**B**) Fraction of the responding rats as a function of contrast. Each point indicates a binary response of an animal to a given contrast, and the resulting distribution was fitted with a probit model (solid line). The 70% confidence interval is outlined with dashed lines. Red lines indicate the ED50 threshold.

Unlike 2 Hz stimulation, animals did not respond to illumination at 20 Hz with constant irradiance (see Suppl. Movie 2), which can be interpreted as flicker fusion or adaptation to continuous stimulus. Therefore, this carrier frequency (or higher) should allow continuous perception of slowly varying irradiance, as opposed to the low frequency stimuli, where each pulse of light elicit a new percept. To assess perceptual contrast sensitivity in this approach, we measured the startling response to slow (0.25 Hz) alternation between 2 illumination levels, which modulated the 20 Hz carrier frequency by adjusting pulse duration between 0 to 12 ms (Fig. 2A). Interestingly, we found significantly lower stimulation thresholds for this burst stimulus (Fig. 1C, blue curve) compared to single pulse response (4.8 ± 0.8 ms instead of 6.7 ± 0.6 ms, p < 0.05 paired t-test), suggesting a cumulative perceptual effect of the high frequency burst stimulation. This observation is consistent with previous reports of integration of the network-mediated stimulation by the RGCs^22^.

The binary responses from the animals to the contrast steps were fitted with a probit density function. From this fit, the 50% probability of response to contrast steps occurred at 12 ± 2% contrast (Fig. 2B). Since the dynamic range of the video camera did not allow us to discriminate between the brighter and dimmer phases of the stimulus at low contrast levels, this threshold represents a combination of responses to positive and negative contrast steps. For 100% contrast, however, we quantified the number of freezing responses to onset and to offset of the stimulus. Over 4 min stimulation, we observed 6.6 ± 1.8 (standard error of mean, s.e.m.) startling events due to negative steps and 9.2 ± 1.2 (s.e.m.) events due to positive contrast steps. Although responses to positive steps were more frequent than to negative ones, the difference did not reach statistical significance (paired t-test, p > 0.05).

### RGC responses to single-pulse electrical stimulation *ex-vivo*

**Figure 3:**
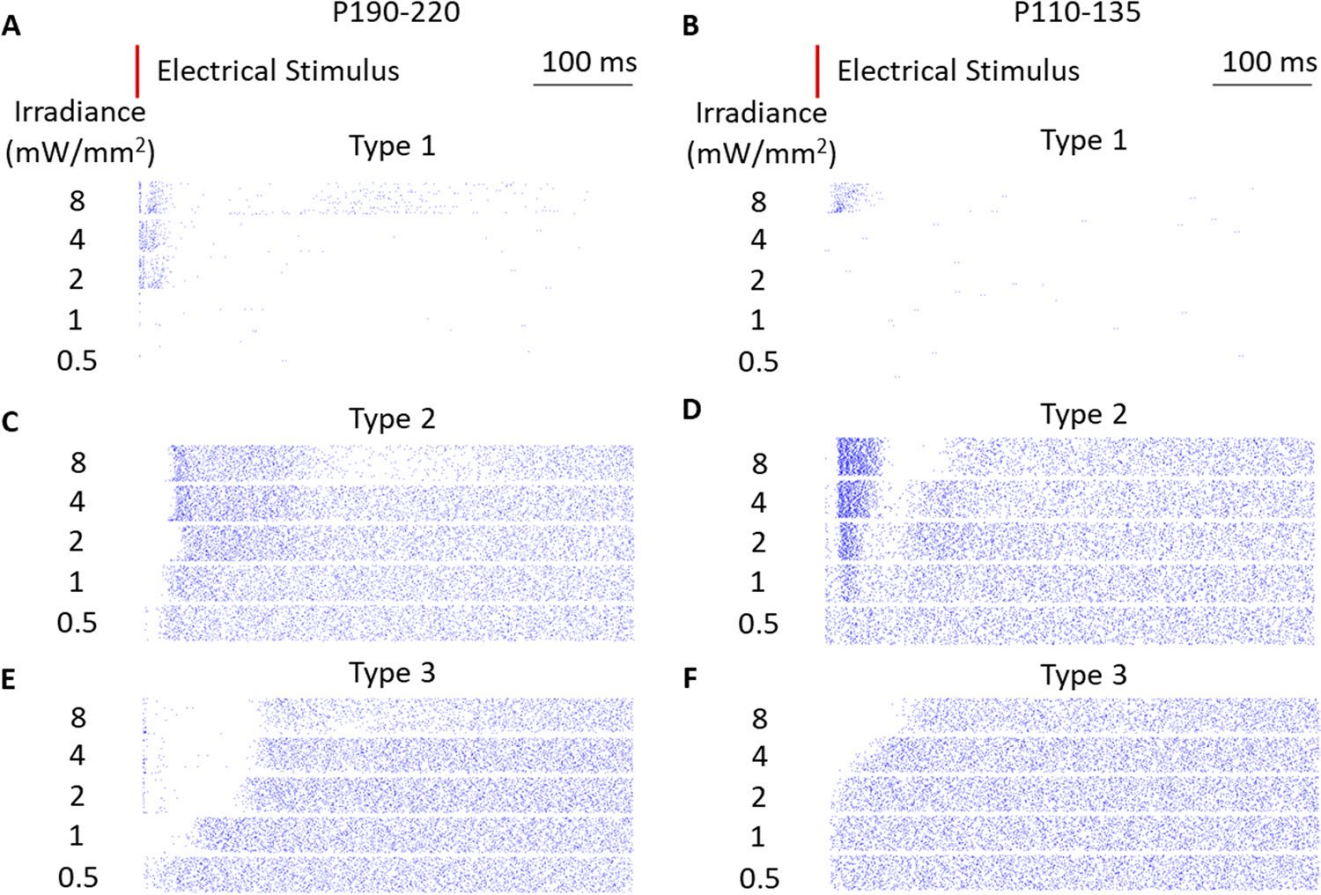
Raster plots of RGC responses to a single electrical pulse in P200 (**A**,**C**,**E**) and P120 RCS rats (**B**,**D**,**F**). (**A**,**B**) Cells with low spontaneous firing rate (36 out of 115 cells) were consistent with previous report^18^. (**C**) The dominant response (49 out of 115 cells) begins with a short-latency inhibition, followed by an excitatory peak. At higher intensities, some cells exhibit a delayed weaker inhibitory phase. (**D**) Younger retinas (P110-135) had stronger excitatory peak, as well as lower threshold and shorter duration of the second inhibitory wave. (**E**) The 3rd type of response (30 out of 115 cells) includes a long inhibition, with its duration increasing beyond 100 ms at high irradiances. (**F**) Largely similar response was observed in less degenerate retina, albeit with slightly lower spontaneous firing rate.

To investigate the mechanisms underlying responses to temporal contrast steps *ex-vivo*, we measured RGC responses to single electrical pulses on MEA, by applying temporally sparse (2 Hz) 4-ms laser pulses to a photovoltaic array placed on top of an RCS retina at various light intensities over *n* = *120* trials. In P190-P220 and P110-P135 rat retinas, we were able to identify cells with low spontaneous firing rate (N = 36, Fig. 3A,B), similar to those we reported previously^18^. In addition, two types of responses could be distinguished in cells with high spontaneous firing levels by their excitatory and inhibitory phases (Fig. 3C,D vs. E,F). The majority of these cells (N = 49) (“type-2 responses”) exhibited a tri-phasic response: two inhibitory phases at high irradiances, including a short-latency complete suppression of spontaneous firing, and a delayed long inhibition, which could last for 250 ms. The first inhibitory phase has a lower stimulation threshold than the following peak. A considerable number of cells had similar responses, but did not feature the delayed inhibition. Younger retinas with less degeneration (P110-135, Fig. 3D) had a stronger excitatory peak, as well as lower threshold and shorter duration of the second inhibitory wave. These cells typically exhibited responses to every stimulus in a 20 Hz pulse train, and therefore were chosen for the contrast sensitivity analysis.

The signature of the other type of response (“type 3”, N = 30, Fig. 3E,F) was its short-latency, long-lasting inhibition that could extend up to 100 ms post-stimulus. This response appeared at irradiances exceeding 1 mW/mm^2^. Since the data during the first 8.25 ms following the electrical pulse was discarded due to electrical artifact (see Methods), shorter latency responses could not be observed.

### RGC responses to repetitive electrical stimulation at 20 Hz

**Figure 4:**
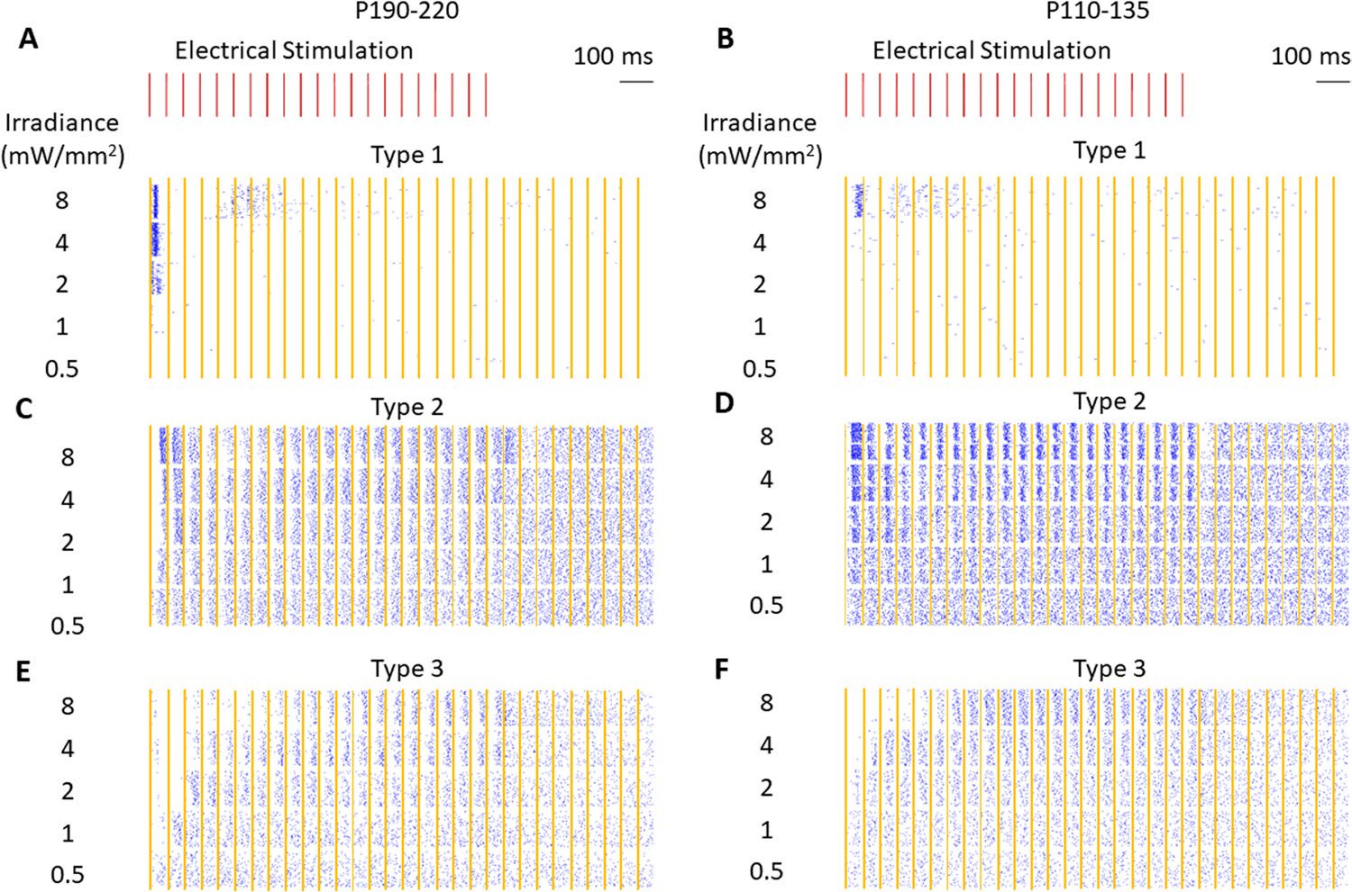
Raster plots of RGC responses to 20-Hz repetitive stimulation for 1 second. Panels directly correspond to the same cells shown in Fig. 3. Orange strips indicate 8.25 ms windows when data was blanked to avoid stimulation artifacts. (**A**,**B**) Cells with type-1 response showed flicker fusion. (**C**) Cells with type-2 response exhibited inhibition and excitation after every pulse, with stronger excitation at the beginning and at the end of the burst. (**D**) Younger retina (P110-135) exhibited stronger excitatory responses. (**E**) When stimulated at 20 Hz, type-3 response included inhibition of spontaneous firing at the beginning of the burst, with duration of the inhibitory period increasing with the stimulus intensity. After that, it reached a steady-state response including inhibitory and excitatory phases after every pulse. (**F**) Younger retina showed similar responses.

Cells responding to a 1 second burst of 4-ms NIR pulses repeated at 20 Hz (equivalent to 100% contrast) were cross-identified using EIs obtained with their single-pulse stimulation, and their responses are shown in Fig. 4. Due to artifact removal, 8.25-ms time intervals are excluded in every 50 ms long cycle, creating empty vertical strips in the raster plot. Cells with low spontaneous firing rate responded only to beginning of the high frequency burst, exhibiting adaptation or flicker fusion at high-frequency pulsing (Fig. 4A,B), similar to previously reported observations^18^. However, cells with high spontaneous firing rate responded to every stimulus (Fig. 4C–F).

In response type-2, the short latency inhibition occurred after every pulse at all measured irradiances (Fig. 4C). Excitatory responses appeared at irradiances above 2 mW/mm^2^. Delayed inhibition appeared at 8 mW/mm^2^, and the timing of maximum inhibition (around the 4^th^ to 5^th^ pulse) aligns with that observed during single-pulse stimulation (Fig. 3A). Excitatory response to the last pulse in the 1-second long stimulation train (Fig. 4C) continued longer than others probably due to lack of inhibition otherwise produced by the next pulse. These cells were responsible for the high contrast sensitivity (low contrast threshold), due to (1) low degree of flicker fusion, so their response is reset after each pulse at carrier frequency, and (2) short-latency peaking pattern.

In response type 3, the first pulse in the train strongly inhibited the cell, desensitizing it for a few following pulses (Fig. 4E,F). In the adapted state, every pulse elicited inhibitory and then excitatory response, as opposed to much longer and predominantly inhibitory response to single pulses (Fig. 3E,F).

**Figure 5:**
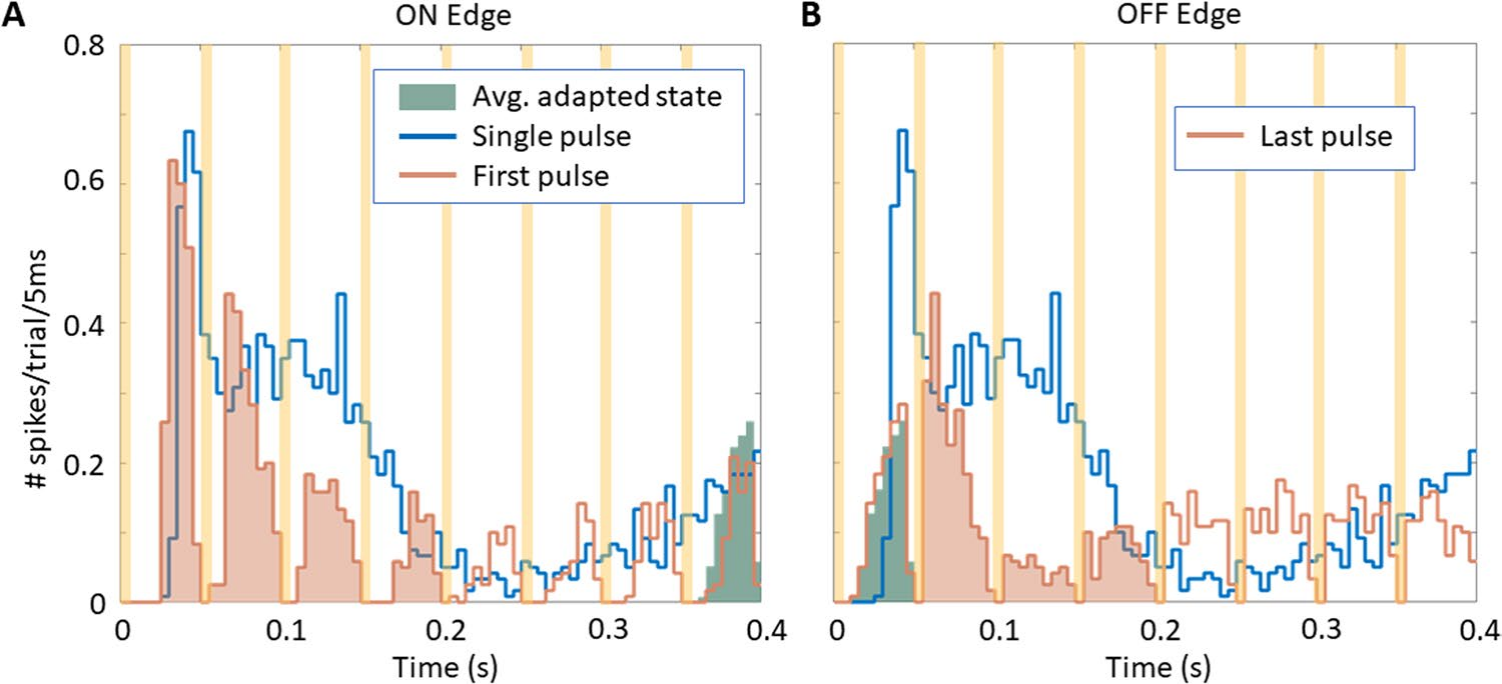
PSTH of a single type-2 cellular response to: (**A**) the first pulse in a high frequency train (orange line), a single electrical pulse (blue line), and 50 ms of the steady-state response during 20 Hz stimulation (green shadow) placed for comparison after the last pulse in the histogram range; (**B**) response to the last pulse of the same train (orange line). The green shadow is now placed after the last pulse of the train, which is at the beginning of the histogram range. The orange shadows illustrate the transient responses prior to returning to the spontaneous firing rate. Stimulus of 8 mW/mm^2^ irradiance with 4 ms pulses at 20 Hz was applied for 1 second. Recording was blocked for 8.25 ms immediately following the onset of each pulse to avoid the stimulation artifact, as indicated by the yellow strips.

**Figure 6:**
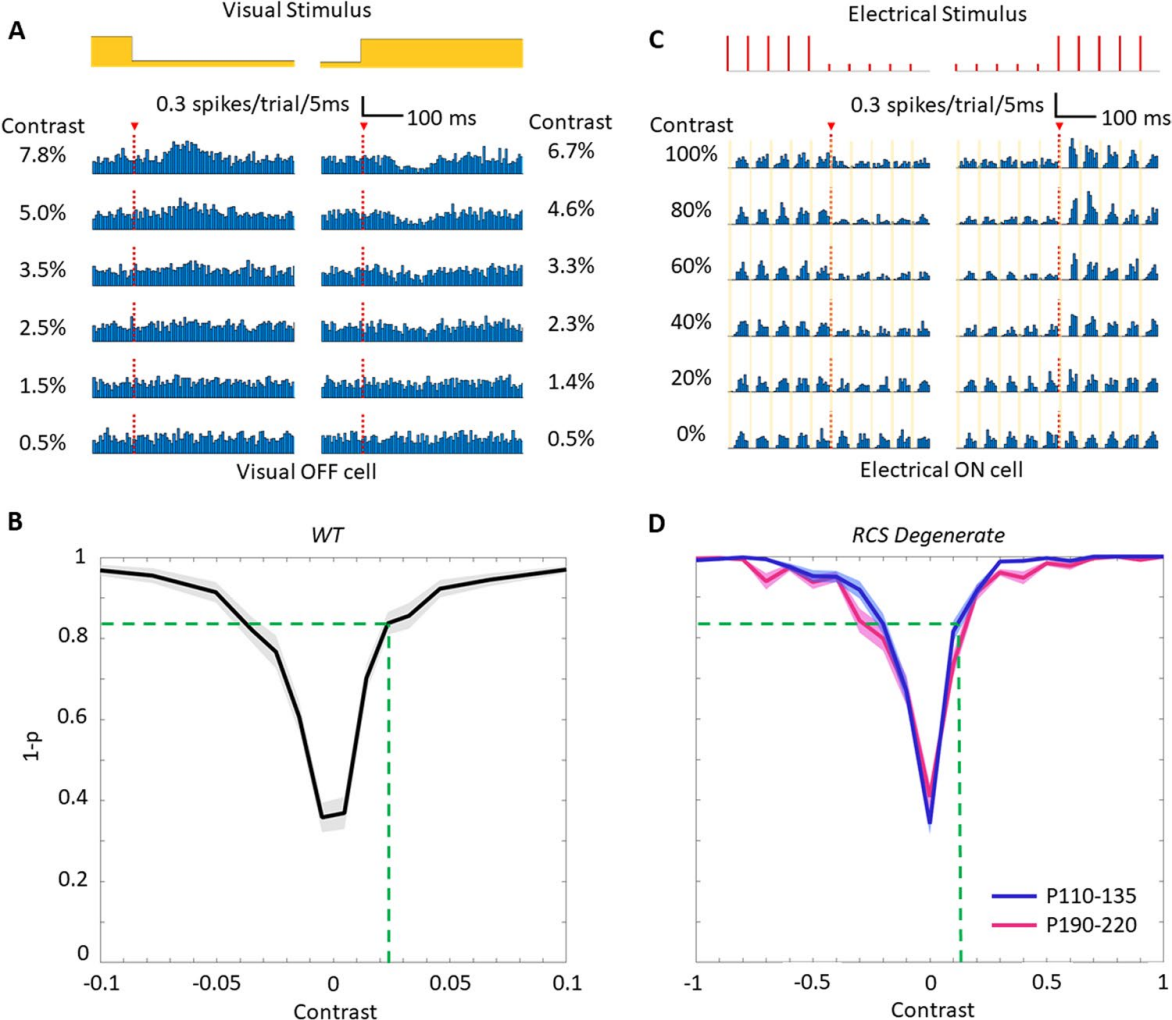
Contrast sensitivity measurements in the healthy and degenerate retina. (**A**) PSTH of a visual OFF RGC responses to 500 ms steps in intensity of the visual stimuli applied to healthy retina. (**B**) Variation of (1 − *p*) value with contrast for visual OFF RGCs. The error band represents one standard error of the mean in the vertical axis. (**C**) PSTH of an RCS RGC responses to 500 ms steps in intensity of photovoltaic stimuli repeated at 20 Hz carrier frequency. Orange strips indicate timings when data was blanked. (**D**) Variation of (1 − *p*)-value with contrast for P110-135 and P190-220 RCS retinas. The green dashed lines show (1 − *p*) values and contrast in natural vision corresponding to the 12% behavioral response threshold. The black dashed lines show (1 − *p*) values and contrast in prosthetic vision corresponding to 2.3% contrast in natural vision.

Steady-state responses to high frequency stimulation differed significantly from responses to a single pulse (Fig. 5). Since each stimulus resets the cell response during repetitive stimulation at 20 Hz, spiking patterns within the 50 ms cycle were shorter and weaker than the response to a single pulse. At the beginning of the train or with increase in the stimulus intensity, cells with type-2 response increased their firing rate after the first pulse, which gradually decreased to the steady-state level with the following pulses (Fig. 5A and top right panel in Fig. 6C). This feature can be considered the ON response to a high frequency stimulation train. Since the response to the last pulse in the train is not affected by the next stimulus, it lasts longer than the steady state, which allows it to reach higher amplitude and larger total number of elicited spikes (Fig. 5B). This unique feature can be considered an excitatory OFF response to the end of the high frequency stimulation train.

### Responses to temporal contrast steps *ex-vivo*

Thresholds of *ex-vivo* responses to changes in full-field illumination were measured by applying either (1) visible light to a WT retina for natural vision, or (2) NIR pulses to a photovoltaic array placed onto an RCS retina for prosthetic vision. For the healthy retina, we projected full-field white light stimuli, varying the irradiance every half a second. OFF cells responded with the lowest contrast threshold (133 cells in 3 retinas). A typical excitatory response to decrease in irradiance and inhibitory response to increase in irradiance are shown in Fig. 6A. Previously, the retinal response to such stimuli was quantified *ex-vivo* using an increase in the spiking rate relative to spontaneous firing^18^ (see Suppl. Figure 2A). To include other aspects of the retinal response, such as inhibitory behavior and spike timing, we applied a new metric: comparing the temporal spike rate distribution within 25–250 ms of a contrast step to that of spontaneous activity (2-sample K-S test). High (1 − *p*) value indicates high likelihood of a statistical difference in temporal distribution of spiking between the two patterns. Under this metric, OFF cells ramp up their response from 0 to 5% contrast, and reach a plateau above 10% contrast (Fig. 6B).

Similar measurements were performed with photovoltaic stimulation of RCS retinas, using 1-second-long bursts of 4-ms pulses at 20-Hz, with variable intensity (see Methods). Only cells that had parts of their soma directly under the implant and exhibited type-2 response were selected for analysis. Noisy cells that showed flicker fusion, in general, exhibited similar or weaker transient responses compared to the selected cells. Incidentally, flicker-fused cells were mostly located near the edge or completely outside of the implant. In other words, they were farther away from the laser beam center than the selected cells. With our selection criteria, most of these flicker-fused cells were naturally excluded, and we decided to ignore the remaining ones under the implant. Quiet cells that were examined previously^18^ were unsuitable for our statistical analysis due to the prevalence of time windows with zero spike rate.

For each contrast step, we compared the spike rate distribution 250 ms before and after the change in irradiance. For high contrasts, an OFF step transitioned a cell from one adapted state to another (~50–100 ms) faster than the ON step (>250 ms) (Fig. 6C). Contrast detection curves, shown in Fig. 6D, were similar for two age groups representing different levels of retinal degeneration: P110–135 (50 cells in 3 retinas) and P190–220 (41 cells in 4 retinas).

Comparison of the temporal contrast sensitivity of natural and prosthetic vision shown in Fig. 6B,D demonstrates that prosthetic responses of degenerate retinas had approximately 5–6 times lower temporal contrast sensitivity than with natural vision: the +12% behavioral response threshold for prosthetic vision defines a critical value (1 − *p*) = 0.84, which corresponds to +2.3% in natural vision. Incidentally, different sensitivity to positive and negative contrast steps observed here with natural vision matches the previously reported asymmetry^23^, and is also present in prosthetic vision. Note that counting just the number of spikes resulted in much higher contrast thresholds (see Suppl. Figure 2).

### Effects of pixilation on contrast detection

**Figure 7:**
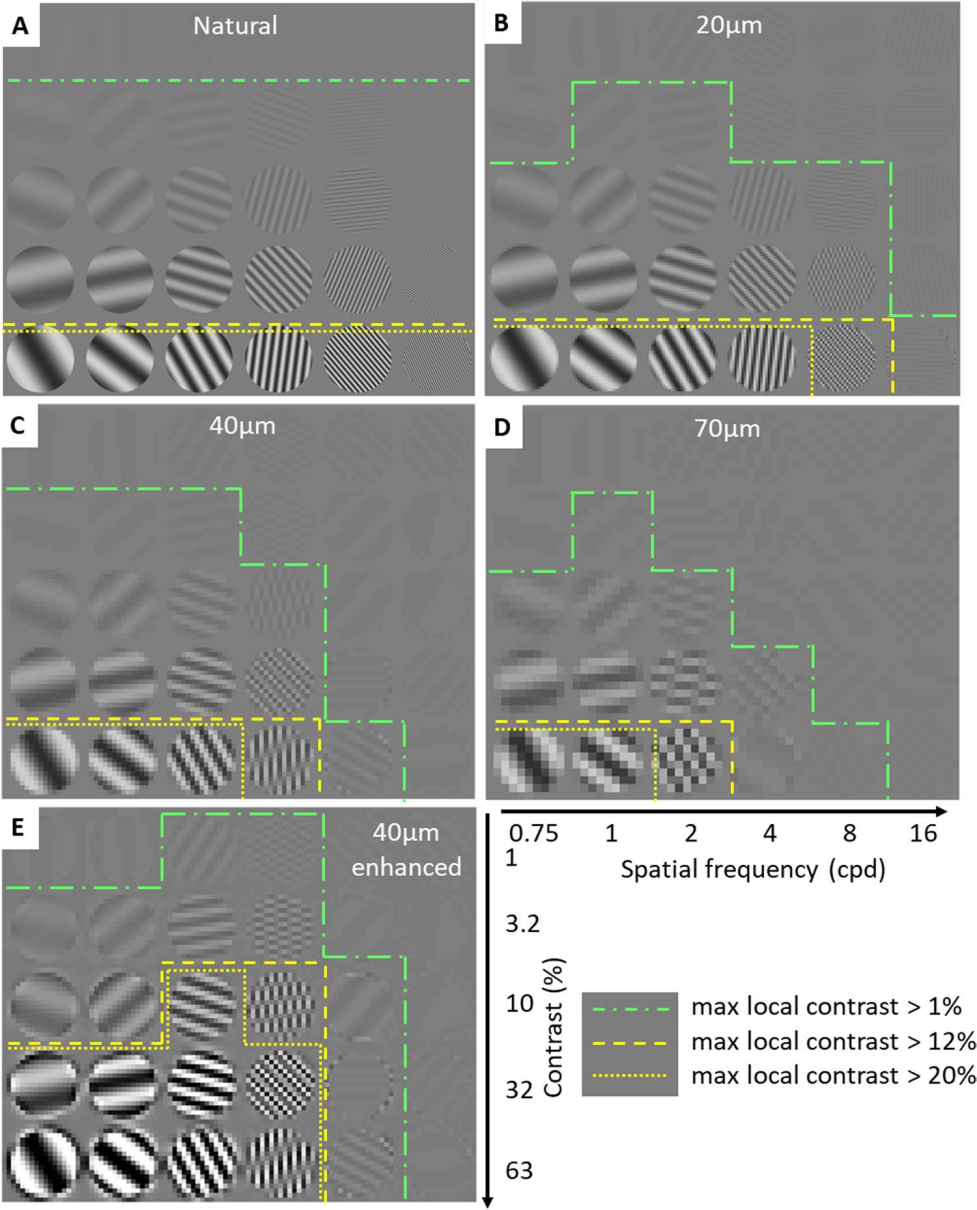
Effect of pixilation and reduced contrast sensitivity on visibility. (**A**) Original image of a FACT-like chart. (**B**–**D**) Pixelated charts for pixel sizes of 20, 40 and 70 µm. (**E**) Contrast is enhanced using unsharp masking prior to pixilation, which increases the number of visible patches within the 12 and 20% contrast sensitivity limits. Areas under the green lines indicate that the maximum local contrast (MLC) of a circular patch exceeds 1%, corresponding to naturally visible levels. Yellow lines indicate patches with MLC exceeding 12% (dashed line) and 20% (dotted line), illustrating the expected limits of prosthetic vision. Scale corresponds to the levels of contrast in the original image.

Reduced contrast sensitivity significantly affects perception of natural scenes. Due to adaptation to the average luminance, local spatio-temporal contrast of a visual stimulus defines visual perception of brightness^24–26^. In humans, the eyes typically fixate for 200–300 ms, and the retina locally adapts to the average luminance^27,28^. Upon ocular movement to another position (such as microsaccades, drift, and ocular tremor), the image on the retina shifts, and retinal ganglion cells respond to the local change in contrast^29^. Both, the implant pixel size and contrast sensitivity provided by the device will therefore impact the patient’s ability to perceive images. We illustrated this effect using sinusoidal gratings of various spatial frequencies and contrast levels, shown in circular patches corresponding to 2° visual angle (Fig. 7). An image sampled by retinal prosthesis becomes pixelated, as shown in Fig. 7B–D. For natural spatial resolution, local contrast within each patch (calculated as described in Methods) remains relatively constant across spatial frequencies. Areas with maximum local contrast (MLC) exceeding 1% are below the green line. Pixilation in prosthetic vision reduces both, spatial resolution and contrast of the high-density patterns. For example, arrays with 40 μm pixels can resolve patches below 2 cpd (150 μm/cycle), while they are unable to properly sample above 8 cpd (37.5 μm/cycle). Intermediate spatial frequencies create heavy aliasing, as exemplified in the patch of 4 cpd at the bottom of Fig. 7C. Decrease in pixel size not only improves spatial resolution, but also increases the average local contrast, as illustrated by the 3 lines outlining the areas of local contrast exceeding 1, 12 and 20%.

Image processing prior to projection onto the photovoltaic array can help improve the contrast of a pixelated image. For example, application of unsharp masking enhances the 40μm-pixelated image, as illustrated in Fig. 7E: with sharpened edges and increased dynamic range, local contrast is increased. This increases the areas visible within the limited contrast sensitivity of prosthetic vision, as outlined by the yellow dash lines. Without image processing, prosthetic vision can only identify 3–4 patches (63% contrast, <4cpd). In the enhanced image, 6 additional patches exceed the contrast sensitivity threshold of prosthetic vision (>10% original contrast).

## Discussion

Both ON and OFF RGC responses to electrical stimulation have been observed previously^30,31^. With full-field stimulation, however, thresholds where previously assessed based exclusively on excitatory responses, i.e. by counting the elicited spikes. On average, positive steps with contrast of +67% were required to elicit an action potential in RGCs with a 50% probability^18^, and no significant excitatory responses to negative contrast steps were reported. These results matched similar thresholds estimated from VEP measurements *in-vivo* – large positive contrast steps are required to elicit significant cortical activity, and no responses to negative contrast steps were observed^13^. It therefore came as a surprise that animals responded behaviorally to prosthetic stimulation not only with an increase, but also a decrease in light intensity, and with contrast steps as low as 12%, in apparent contradiction with previous analyses of RGC responses.

However, a growing body of evidence points to the importance of the millisecond-precision timing of action potentials in sensory inputs to the brain^32–34^, and raster plots of the steady-state retinal response to high frequency stimulation demonstrate that even though there is little difference in the average firing rate of retinal ganglion cells, the temporal structure of their spiking patterns is markedly different. We therefore developed a different cell-selection criteria and analysis method for the recorded spike trains to better distinguish between spontaneous and elicited firing patterns in the absence of change in the average firing rate. While cells with low spontaneous firing rate and flicker fusion were found consistently, they were less sensitive to contrast steps, and could not explain the lower temporal contrast threshold we observed *in-vivo*. The cell types selected in our current analysis had higher spontaneous firing rate and did not exhibit flicker fusion, but they preserved the variation of elicited spike counts with contrast steps, as reported previously^18^, and showed temporally well-structured responses suitable for statistical analysis. The 2-sample K-S test was used to evaluate how much the temporal structure of the response changes (because of the time-dependent increases or decreases in the spiking rate, or a combination thereof) and to detect a change in response pattern even when, on average, no additional action potentials are elicited. Behavioral responses of RCS rats to prosthetic stimulation, where contrast was modulated by pulse duration rather than by amplitude, yielded perceptual thresholds of approximately 12%. Since this perceptual threshold was measured without discriminating between the ON and OFF contrast steps, it corresponds to the highest sensitivity curve in *ex-vivo* measurements, which was a response to positive contrast steps. From our *ex-vivo* analysis, the 12% prosthetic contrast sensitivity corresponds to the same (1 − *p*) value as with a 2.3% visual contrast in healthy retinas, which agrees with 2–3% contrast sensitivity observed in Y-RGCs in rat retinas using drifting gratings^35^. Prosthetic contrast sensitivity was 5–6 times worse than natural at all contrast levels, and it remains unknown whether positive and negative contrast steps in prosthetic stimulation were perceptually distinct in animals that responded behaviorally to both onset and offset of light. Lack of response to prosthetic stimulation in WT rats indicates that 1) the infrared stimulation does not elicit any visual response or 2) that the prosthetic stimulation may be competing with normal vision – as predicted by earlier VEP measurements^36^.

Previously, we described how cells with low spontaneous firing rate exhibit flicker fusion and high spatial resolution in response to alternating gratings due to non-linear summation of the sub-units in the RGC receptive fields, unlike the cells that did not exhibit flicker fusion^14,18^. Interestingly, these flicker-fused cells (response type-1 in the current study) did not provide high temporal contrast sensitivity^14,18^. On the other hand, cells without flicker fusion (response type 2 and 3), which did not provide high spatial resolution in the previous study, turned out to exhibit higher contrast sensitivity. These observations illustrate how different cells carry different aspects of the visual function in the retina. It remains unclear whether the different types of responses we observed with prosthetic vision map to different anatomical cell types.

Mechanistically, we noticed that the RGC response to repeated stimulation significantly differs from that to a single pulse response (Fig. 5). During repetitive stimulation at 20 Hz, each stimulus appears to reset the cell response, so that the spiking patterns within the 50-ms cycle are shorter and weaker than the response to a single pulse. Response resetting was previously reported for ON RGCs in healthy rabbit retinas^37^, and we also observed this phenomenon here with subretinal prosthetic stimulation. Response to the beginning of the train (or to increase in the stimulus intensity) represents a transition from a much weaker adapted state to a strong response to the first pulse, gradually diminishing to a new steady-state (top right panel in Fig. 6C), which can be considered the ON response to a high frequency stimulation train. Response to the last pulse in the train is not affected by the next stimulus, and therefore it lasts longer (Fig. 5), which can be considered an excitatory response to decrease in illumination (OFF response).

Implant pixel size limits not only spatial resolution but also contrast sensitivity, as demonstrated in Fig. 7. Spatial features smaller than the pixel size are unresolvable, and contrast is weak for slightly larger features. Contrast can be improved through image processing for spatial features larger than the pixel size. Unsharp masking accentuates edges, in addition to increasing image contrast. Filter parameters should be carefully chosen to balance between enhancing the contrast and maintaining sufficient number of grey levels (with 12% contrast sensitivity, we might expect about 8 resolvable levels of grey). For example, the bottom row of Fig. 7E demonstrates over-sharpening: patches become binary, and, therefore, we cannot differentiate between sinusoidal and square patterns. The same sharpening, however, is appropriate for lower contrasts in the same chart, where intermediate shades of light are preserved. More sophisticated methods^38,39^ may be explored for optimal image processing.

## Conclusions

Behaviorally, animals with photovoltaic subretinal implants responded to both increase and decrease in intensity of high frequency (20 Hz) stimulation, with a contrast threshold of 12% (Michelson). *Ex-vivo*, retinal ganglion cells with high spontaneous firing rate in the degenerate retina exhibited inhibitory and excitatory responses to both positive and negative contrast steps. The behavioral response threshold could not be explained only through spike counting, and instead better matched the threshold defined by the millisecond-precision timing of RGC spiking, especially in regimes of low contrast steps, where spike counts did not change significantly. The difference in response to the beginning and the end of a pulse train may be interpreted as the prosthetic ON and OFF responses to high frequency stimulation. Temporal contrast steps of 12% in prosthetic stimulation, applied for 1 second at 20 Hz carrier frequency, elicited responses comparable to that of 2.3% steps with visual stimulation in healthy retinas. Contrast enhancement between the camera and prosthetic array could partially compensate for the reduced contrast sensitivity of prosthetic vision.

## Methods

### Photovoltaic implants

The 1 and 2 mm-wide arrays of 30 µm in thickness with 3-diode photovoltaic pixels of 70, 140 and 280 µm in size were manufactured according to previously described methods^40^, except for reversal of the n- and p-doped regions to produce anodic-first pulses. *Ex-vivo*, we used arrays of 1 mm in width with 70-µm pixels. *In-vivo* though, in order to enable operation at lower light intensity and with wider illumination angles, we used 1 and 2 mm wide implants with 140 µm and 280 µm pixels. All light stimuli used in this study are free of spatial content, making it possible to compare results obtained with devices with different pixel sizes.

### Animals

Rats with retinal degeneration were obtained from a Royal College of Surgeons (RCS) colony maintained at the Stanford Animal Facility. Female Long-Evans adult wild type (LE, WT) rats were purchased from Charles River (Wilmington, MA, USC). All animals were housed in a 12-hour light/12-hour dark cycle with food and water *ad libitum*. All experimental procedures were approved by the Stanford Administrative Panel on Laboratory Animal Care, and conducted in accordance with the institutional guidelines and conformed to the Statement for the Use of Animals in Ophthalmic and Vision research of the Association for Research in Vision and Ophthalmology (ARVO).

### *In-vivo* stimulation

Photovoltaic arrays were implanted subretinally in rats (RCS, P105-120, n = 6; WT, P90, n = 3), as described previously^12,14^. Animals were anesthetized (IM) with a mixture of ketamine (75 mg/kg) and xylazine (5 mg/kg). After application of topical anesthetic, the superior part of the sclera was exposed and a 2 mm incision through the sclera/choroid/retina was made 1.5 mm from the limbus. The retinal incision was necessary to allow proper retinal detachment without increase in intraocular pressure. After lifting the retina with balanced saline solution (BSS), the implants were inserted into the subretinal space and the incision was closed with a 10–0 nylon suture. Topical antibiotics (Bacitracin/PolymyxinB) were applied, and the animals were recovered. Over the course of one week, photoreceptor outer segments above the implant degenerated, followed by a gradual disappearance of the outer nuclear layer due to permanent separation from the pigment epithelium^41^.

For behavioral measurements, dilation drops (tropicamide/phenilephrine) were applied 15 min prior to the procedure. Animals were placed into a cage surrounded by 4 or 6 near-infrared (850 nm) LED panels (CMVision-IR200) and monitored by a video camera (Fig. 1A). Animals were habituated to the cage for 4 min before stimulation started. A freezing event was defined as no motion other than breathing for more than 2 seconds. For the stimulation threshold measurements, light pulses were applied at 2 Hz for 30 seconds at each level of irradiance (0.001–0.58 mW/mm^2^), and pulse duration (1–10 ms). Behavioral responses recorded on the video were analyzed offline by the experimenters.

For contrast sensitivity measurements, light with 0.58 mW/mm^2^ incident irradiance on the rat’s eye was applied at 20 Hz carrier frequency for 3 minutes with 0.25 Hz alternation between low and high average irradiance, modulated by pulse width (Fig. 2A). We verified that with the illumination system used, eye irradiance was independent of the animal’s head position. Contrast steps were quantified using the Michelson definition, i.e. contrast $C\,=\,({I}_{max}-{I}_{min})/({I}_{max}+{I}_{min})$, where ${I}_{max}$ and ${I}_{min}$ are the average maximum and minimum radiant exposures during the burst, respectively. For each animal, ${I}_{min}$ was chosen as the stimulation threshold, since otherwise the effective contrast would be 100%, rather than the calculated value for $C$. As a result of variation in the stimulation thresholds (<20% s.e.m.), the same radiant exposures represented slightly different contrast values for each animal. This binary distribution was fitted with a probit model, with a 70% confidence interval. Thresholds and their variability were defined as the intersection of the fitted curve and confidence interval curves with the 50% probability line.

### Electrophysiological recordings *ex-vivo*

Eyes were enucleated from euthanized (390 mg/kg pentobarbital sodium, 50 mg/mL phenytoin sodium) rats (RCS P190-220, n = 4, RCS P110-135, n = 3 and WT n = 3). A small piece of retina (~3 mm × 3 mm) was isolated and placed ganglion cell side down on a 512-electrode multielectrode array (MEA)^17^. The retina was constantly perfused with Ames’ medium at 29.4 °C and bubbled with a mixture of 95% O_2_ and 5% CO_2_. For assessment of prosthetic vision, a photovoltaic implant was placed on top of the retina, mimicking a subretinal placement *in-vivo*^8^. We used a nylon mesh (~100 μm cell size) to lightly press the implant and retina onto the MEA for better contact. The same procedures were undertaken for natural vision without the implant in place. Voltage waveforms from each of the 512 electrodes on the MEA were amplified and digitized with 20 kHz sampling frequency^17^.

For prosthetic vision, an 880-nm diode laser coupled via a 400-μm multimode fiber was used for illumination. The beam exiting from the fiber was collimated, homogenized using a 2° divergence microlens array diffuser, and projected onto the implant via the camera port of an inverted microscope. The projection system was calibrated to deliver a maximum peak irradiance of 8 mW/mm^2^ onto the sample. For contrast sensitivity measurements, we used a carrier waveform consisting of 4-ms square NIR pulses applied at 20 Hz. Contrast steps were constructed by modulating the amplitude (peak power) with 0.5-second-long phase of 8 mW/mm^2^, followed by a 0.5-second-long phase of a chosen lower light intensity, and then back to 8 mW/mm^2^. We used *n* = *80* trials for every contrast step. For single-pulse stimulation, 4-ms square NIR pulses were applied at 1 Hz repetition rate, and *n* = *120* trials were recorded to determine the RGC responses. For measurements of the stimulation thresholds at high frequency, as well as the ON and OFF responses to 100% contrast steps, we used a similar paradigm to contrast sensitivity tests, but the laser was turned off during the dark phase, and we varied the irradiance during the bright phase. Each alternating phase lasted for 1 second, and *n* = *120* trials were recorded.

For natural vision, the image of a 15″ CRT screen was optically reduced in size and projected onto the photoreceptor layer of a healthy retina through the camera port of an inverted microscope. Modulation of light intensity was performed with 0.5-second-long steps, similar to the envelop of prosthetic stimulation. To differentiate between ON- and OFF-center RGCs in WT retinas, we applied spatiotemporal monochromatic white noise using 70 × 70 μm^2^ pixels refreshed every 33 ms^42^.

### Data analysis for recordings *ex-vivo*

Artifacts in the raw recording traces caused by prosthetic stimulation were first removed from the recordings. For every individual electrical pulse and electrode, we estimated the artifact by fitting a 7^th^-order polynomial to the data between 8.25 ms and 50 ms following the onset of the pulse. The fitted polynomial was then subtracted point-wise from the raw voltage trace. During the first 8.25 ms following the onset of each pulse, the artifact saturated the recording amplifiers and therefore could not be successfully subtracted from the raw voltage trace. As a result, action potentials (spikes) elicited during that period were discarded. Supplementary Figure 1 shows an example of a voltage trace recorded on one of the electrodes before and after the artifact subtraction.

The artifact-subtracted traces were then used for spike detection and sorting using custom software^17^. Spikes were defined as an event where the negative voltage deflection amplitude exceeded 4.5 times the root-mean-square (RMS) noise on each electrode. We applied dimensionality reduction to the detected spike waveforms using principal component analysis, followed by expectation-maximization clustering^17^. For each putative neuron, we calculated the electrophysiological image (EI) of the neuron - the average electrical signal measured on the whole multielectrode array when the neuron produced an action potential. It typically shows the soma location and axonal trajectory of the RGC^43,44^. In our analyses, we excluded neurons with abnormal EIs, such as the ones involving backpropagating or multi-axonal action potentials. Location of RGCs somas was estimated from their EIs^45^, and only RGCs with somas completely or partially under the implant were kept for further analysis. Their responses to prosthetic stimulation were manually classified according to their raster response properties. For visible light stimulation, cells were classified as ON- and OFF-center types based on polarity of the first peak in the spike-triggered average (STAs) traces.

Similar to *in-vivo* measurements, we used the Michelson definition for contrast $C=({I}_{post}-{I}_{pre})/({I}_{post}+{I}_{pre})$, where ${I}_{pre}$ and ${I}_{post}$ are the luminances (or peak irradiance for prosthetic stimulation) preceding or following the contrast step, respectively. To assess cellular response to prosthetic stimulation, we compared the distributions of spike rates during 250 ms before and after a contrast step. We selected cells whose EIs fully or partially overlap with the implant and elicited periodic responses to every pulse in a 20 Hz electrical pulse train. The spike rates were computed by convoluting spike times with a 5-ms rectangular temporal filter, the width of which was chosen for optimal resolution of PSTHs for Poisson point processes^46^,^47^. The resulting distributions were compared using the two-sample Kolmogorov-Smirnov test. For visible light stimulation, the firing pattern of each RGC during the period from 25 ms to 275 ms post contrast step was compared to its spontaneous firing, using the same statistical method as for electrical stimulation. The 250-ms analysis period was shifted by 25 ms because the phototransduction process is slower than the prosthetic response, and hence RGC responses are delayed compared to electrical stimulation. With this time shift, we captured more delayed responses without compromising earlier spikes, yielding more sensitive contrast detection.

### Visual Representation of Spatial Resolution and Contrast Sensitivity

To illustrate the effect of the prosthesis pixel size on visual perception, we applied a simple computational model to a chart similar to the Functional Acuity Contrast Test (FACT)^48^. Sinusoidal gratings of various spatial frequencies and contrast levels are shown in circular patches, each corresponding to 2° visual angle (Fig. 7A).

For enhancement of prosthetic vision, we performed image sharpening on the chart using unsharp masking as defined in MATLAB (R2015b, The MathWorks Inc., Natick, MA). Three steps were involved in unsharp masking: (1) convolve an image with a 2D Gaussian filter (radius $R$), (2) subtract the filtered image from the original image to obtain an “unsharp mask”, and (3) add the unsharp mask (weighted by amount $A$) to the original image. For our example image in Fig. 7E, we chose $R=40\,\mu m$ and $A=7$.

The chart was pixelated by taking the unweighted mean luminance for each individual pixel. To include the effect of ocular fixational motion, local luminance $L$ and contrast $C$ were averaged over 1° visual angle (“patch”), and were computed using the definitions^49^:

1 $$L=\sum _{i=1}^{N}{w}_{i}{L}_{i}$$

2 $$C=\sqrt{\sum _{i=1}^{N}{w}_{i}{(\frac{{L}_{i}-L}{{L}_{i}+L})}^{2}}$$

where ${L}_{i}$ is the luminance of the $i$-th pixel in a patch with a total $N$ pixels. The weights ${w}_{i}$ were generated from a circularly symmetric raised cosine:

3 $${a}_{i}=\,\cos (\frac{2\pi }{d}\,\sqrt{{x}_{i}^{2}+{y}_{i}^{2}})+1$$

4 $${w}_{i}=\frac{{a}_{i}}{{\sum }_{i=1}^{N}{a}_{i}}$$

where $({x}_{i},\,{y}_{i})$ are the horizontal and vertical distances of the $i$-th pixel away from the center of the patch.

## Electronic supplementary material

Supplementary Materials

Supplementary Movie 1

Supplementary Movie 2
