# Supplementary material for: Temporal structure in spiking patterns of ganglion cells defines perceptual thresholds in rodents with subretinal prosthesis
Source: Sci Rep. 2018 Feb 16;8:3145. doi: 10.1038/s41598-018-21447-1 (PMC5816604; doi:10.1038/s41598-018-21447-1)
Supplement: Supplementary file 1 — Supplementary Materials [file 41598_2018_21447_MOESM1_ESM.pdf]

Temporal structure in spiking patterns of ganglion cells defines perceptual thresholds in rodents with subretinal prosthesis

Elton Ho[1*], Henri Lorach[2,3], Georges Goetz[2,4], Florian Laszlo[2], Xin Lei[5], Theodore Kamins[5], Jean-Charles Mariani[2], Alexander Sher[6], and Daniel Palanker[2,3]

[1]Department of Physics; [2]Hansen Experimental Physics Laboratory; [3]Department of Ophthalmology; [4]Department of Neurosurgery; [5]Department of Electrical Engineering, Stanford University, Stanford, CA 94305, USA
[6]Santa Cruz Institute for Particle Physics, University of California, Santa Cruz, CA 95064, USA

Supplementary Materials

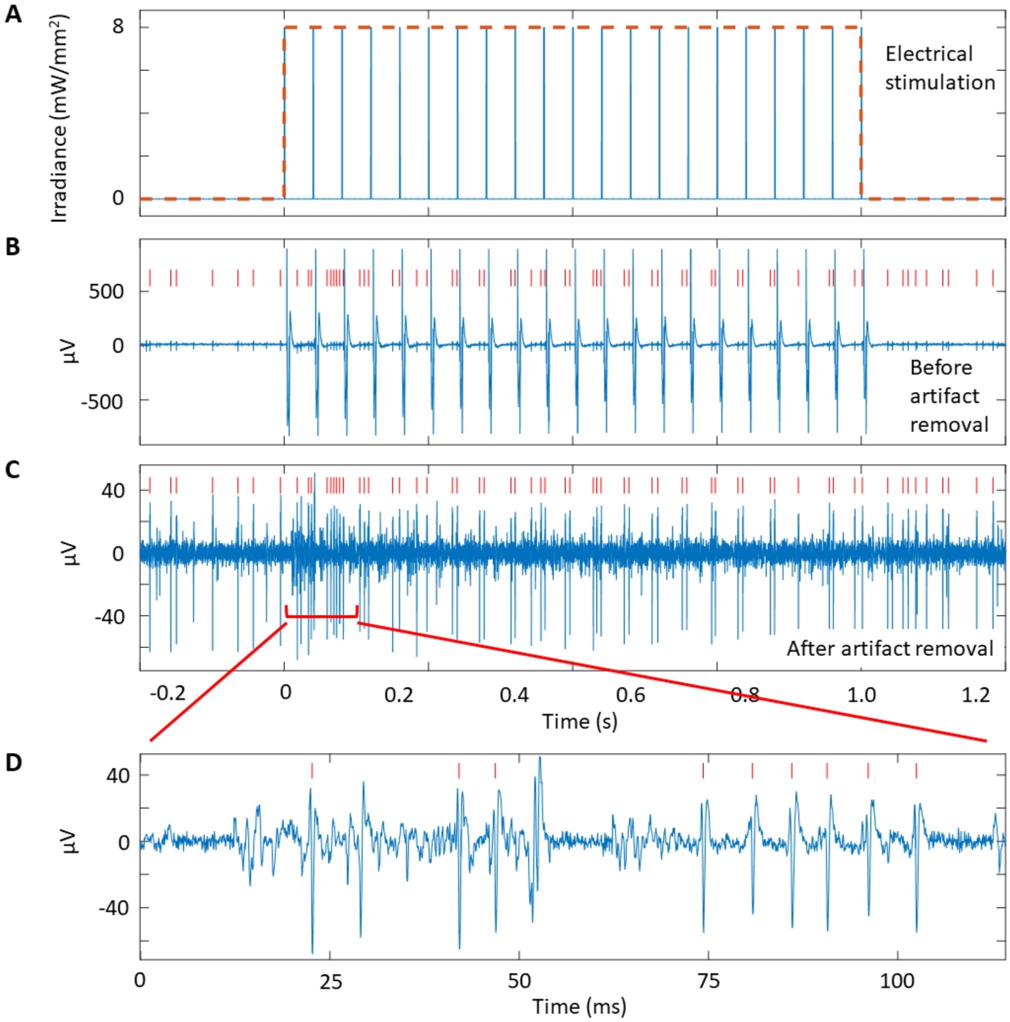

Supplementary Figure 1. Electrical stimulation and recorded waveforms on MEA. (A) NIR pulse train projected onto the photovoltaic implant. (B) Raw trace from an electrode under the implant. Red ticks on top of the traces indicate identified spikes for a cell exhibiting response type 2. (C) Same trace after removing the electrical artifacts. (D) Burst of spikes immediately following the onset of a 100% contrast step.

* Correspondence and requests for materials should be addressed to E.H. (eltonho@stanford.edu)

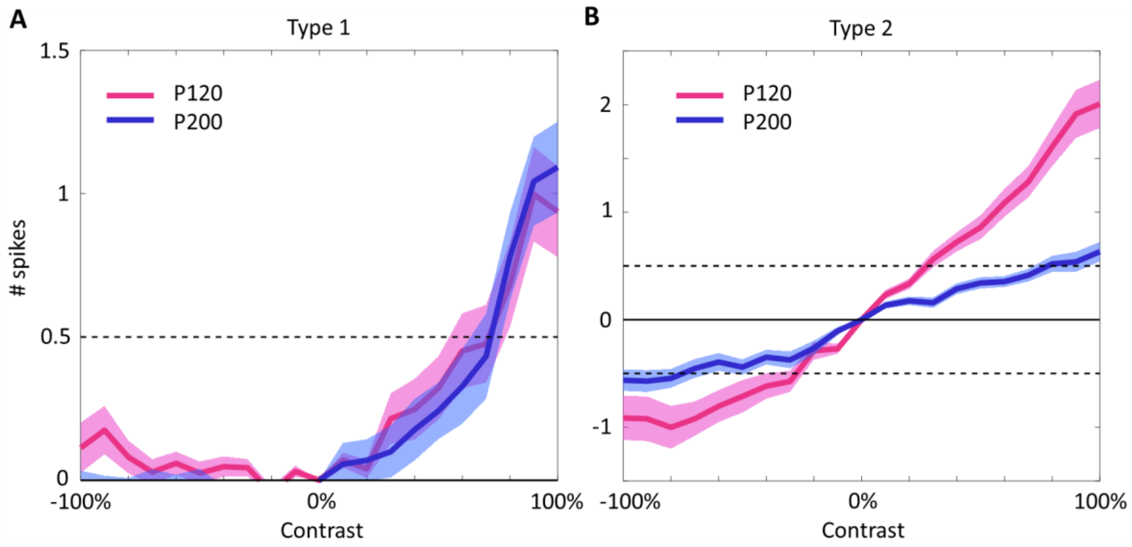

Supplementary Figure 2. Changes in the number of spikes cannot fully account for the low contrast threshold observed in-vivo. (A) Change in spike counts for type-1 cells (low spontaneous firing rate). This data matches the previously reported values *(Goetz et al 2015)*, which represents a much higher contrast threshold (>65%) than reported in the current study (~12%). (B) Change in spike counts for cells with high spontaneous firing rate (type-2), for p120 and p200 RCS rat retinas. For both cell types, counting the number of spikes yields thresholds higher than observed in the current study.

Supplementary Movie 1. RCS rat with a subretinal photovoltaic array responds to 880nm light emitted by the LED panels. As soon as the pulsing begins, the rat jumps, freezes, and later moves into the dark corner of the cage.

Supplementary Movie 2. Adaptation to 20Hz continuous stimulation.
